# Group based trajectory modeling identifies distinct patterns of sympathetic hyperactivity following traumatic brain injury

**DOI:** 10.21203/rs.3.rs-4803007/v1

**Published:** 2024-09-02

**Authors:** Sancharee Hom Chowdhury, Lujie Karen Chen, Peter Hu, Neeraj Badjatia, Jamie Erin Podell

**Affiliations:** University of Maryland Baltimore County; University of Maryland Baltimore County; University of Maryland School of Medicine; University of Maryland School of Medicine; University of Maryland Medical Center

**Keywords:** Brain injuries, traumatic, Autonomic nervous system diseases, Cluster analysis

## Abstract

**Background:**

Paroxysmal Sympathetic Hyperactivity (PSH) occurs with high prevalence among critically ill Traumatic Brain Injury (TBI) patients and is associated with worse outcomes. The PSH-Assessment Measure (PSH-AM) consists of a Clinical Features Scale (CFS) and a Diagnosis Likelihood Tool (DLT), intended to quantify the severity of sympathetically-mediated symptoms and likelihood that they are due to PSH, respectively, on a daily basis. Here, we aim to identify and explore the value of dynamic trends in the evolution of sympathetic hyperactivity following acute TBI using elements of the PSH-AM.

**Methods:**

We performed an observational cohort study of 221 acute critically ill TBI patients for whom daily PSH-AM scores were calculated over the first 14 days of hospitalization. A principled group-based trajectory modeling approach using unsupervised K-means clustering was used to identify distinct patterns of CFS evolution within the cohort. We also evaluated the relationships between trajectory group membership and PSH diagnosis, as well as PSH DLT score, hospital discharge GCS, ICU and hospital length of stay, duration of mechanical ventilation, and mortality. Baseline clinical and demographic features predictive of trajectory group membership were analyzed using univariate screening and multivariate multinomial logistic regression.

**Results:**

We identified four distinct trajectory groups. Trajectory group membership was significantly associated with clinical outcomes including PSH diagnosis and DLT score, ICU length of stay, and duration of mechanical ventilation. Baseline features independently predictive of trajectory group membership included age and post-resuscitation motor GCS.

**Conclusions:**

This study adds to the sparse research characterizing the heterogeneous temporal trends of sympathetic nervous system activation during the acute phase following TBI. This may open avenues for early identification of at-risk patients to receive tailored interventions to limit secondary brain injury associated with autonomic dysfunction and thereby improve TBI patient outcomes.

## Introduction

Paroxysmal Sympathetic Hyperactivity (PSH) is a condition characterized by excessive and uncontrolled activity of the autonomic nervous system, presenting as recurrent episodes of unregulated sympathetic responses including rapid heart rate, increased body temperature, high blood pressure, rapid breathing, muscle stiffening, and excessive sweating[1–3]. The exact cause of PSH is not fully understood, but is likely related to brain injury-related disrupted regulation of the autonomic nervous system. About 80% of reported PSH cases occur in patients with traumatic brain injury (TBI)[3–6], and its prevalence among TBI patients is estimated to be between 8% and 33%, often correlating with poorer outcomes, even when controlling for injury severity[1, 7]. Therefore, early diagnosis and treatment of PSH may improve patient outcomes[1, 2, 8–10].

In 2014, a group of experts proposed the PSH-Assessment Measure (PSH-AM), a diagnostic and monitoring tool designed for adult patients in both clinical and research settings[1]. The PSH-AM consists of two components: the Clinical Feature Scale (CFS), which quantifies the severity of PSH symptoms, and the Diagnostic Likelihood Tool (DLT), which tallies the number of diagnostic criteria met. While the PSH-AM was designed for serial use, only a few studies have characterized its evolution over time in brain injured patients. Among these is an age- and severity-matched case-control study of acute TBI patients from our group, which demonstrated similar CFS scores during the first six post-injury days, followed by higher CFS scores in cases compared to controls during days seven through ten[7]. This finding raises the question of whether more nuanced patient groupings based on CFS trajectory patterns exist and whether they could lead to earlier recognition of post-TBI dysautonomia.

Our focus in this paper is to explore post-TBI physiologic trajectory groups in an unsupervised manner, using a principled group-based trajectory modeling (GBTM) approach[11]. Numerous studies relevant to neurocritical care have employed GBTM to discover distinct physiological trend patterns and assess the predictive value of group membership in relation to clinical outcomes of interest[12–16], but this is the first application of GBTM to understand the evolution of post-TBI dysautonomia. We hypothesize that this data-driven approach may better identify naturally occurring post-TBI physiological phenotypes than one relying on a subjective clinical diagnosis of an imperfectly defined syndrome (e.g., PSH) that occurs along a spectrum of certainty and severity. Identified trajectory groups will form the basis of autonomic endotypes, which we will further characterize by their associated clinical and demographic risk factors and outcomes of interest.

## Methods

### Patient Cohort

**Figure 1:**
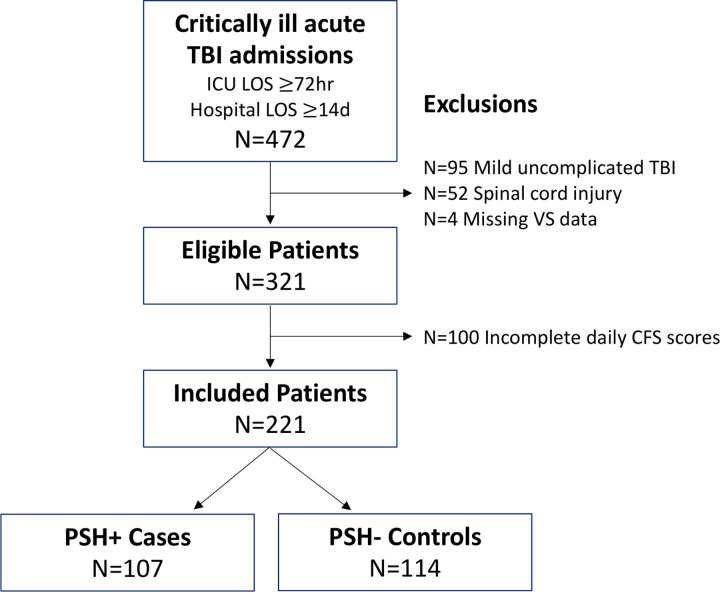
Patient flow diagram Patient flow diagram illustrates the inclusion and exclusion criteria for eligible patients. From 472 critically ill adult TBI patients who were admitted to R Adam Cowley Shock Trauma Center between January 2016 and July 2018, we selected patients who remained in the ICU for at least 3 days and in the hospital for at least 14 days. We excluded those with mild uncomplicated TBI (GCS 13–15 with negative head CT) concomitant spinal cord injury, or incomplete data, resulting in a final cohort of 221 patients. TBI: traumatic brain injury; ICU: intensive care unit; LOS: length of stay; VS: vital sign; CFS: clinical feature score of the paroxysmal sympathetic hyperactivity (PSH) – assessment measure.

This is a retrospective cohort study of 472 critically ill adult TBI patients admitted to R Adams Cowley Shock Trauma Center between January 2016 and July 2018. These eligible patients were identified by institutional trauma registry-adjudicated Head Abbreviated Injury Score (AIS) greater than or equal to 1, with trauma ICU length of stay of at least three days and hospital length of stay of at least 14 days. We further excluded patients with mild uncomplicated TBI [Glasgow Coma Scale (GCS) at ICU admission ≥ 13 and negative head CT] due to lower risk of PSH and those with concomitant spinal cord injury due to potential for spinally-mediated autonomic dysfunction. Patients were also excluded if recorded continuous vital sign data was not available. Of the remaining 321 patients, a convenience sample of 221 who underwent PSH-AM scoring were included in this study (Fig. 1). Demographic and clinical characteristics of the held out sample (N=100) are available for comparison in Supplementary Table 1. As in previous work, PSH positive (case) patients were identified by reviewing medication administration records for institutional first-line PSH pharmacotherapies including bromocriptine and propranolol, and this diagnosis was corroborated by review of clinical documentation from the electronic health record (EHR)[7].

### Calculation of Daily PSH-AM scores

**Figure 2:**
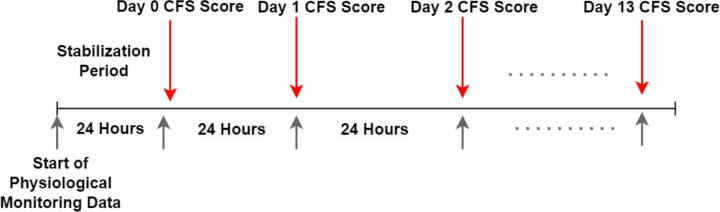
Daily Clinical Features Score calculation from Electronic Health Record data Illustration of daily clinical features score (CFS) score computed from the electronic health record. The Day 0 CFS was excluded from our analysis as the initial 24 hours was considered a stabilization and resuscitation period. CFS: Clinical feature score of the paroxysmal sympathetic hyperactivity – assessment measure.

The PSH-AM score consists of two subscores: the Clinical Feature Scale (CFS) and the Diagnostic Likelihood Tool (DLT)[1]. The CFS assigns a score ranging from 0 to 3 to each category, including heart rate, respiratory rate, blood pressure, temperature, and the presence of sweating and posturing. The overall CFS score, which ranges from 0 to 18, indicates the severity of PSH symptoms for that day, with 0 signifying no symptoms and 18 indicating severe symptoms. Each patient’s daily CFS was computed from vital signs documented in the EHR, based on the highest value observed during each 24-hour window, as illustrated in Fig. 2. For sweating and posturing, scores were allocated based on review of daily clinical notes. Due to lack of more granular information regarding severity of sweating and posturing, a score of 2 (moderate) was given for any mention and 0 (absent) if not specifically noted, as in van Eijck, et al[17]. We considered 13 consecutive days of data after the initial 24-hour resuscitation and stabilization period (day 0).

The DLT score comprises a set of 11 binary indicators, assessing factors such as the simultaneity of clinical features, persistence of features, frequency of episodes, medication administration, and exclusion of other causes of symptoms[1]. Because the DLT characterizes the overall likelihood of PSH diagnosis, rather than physiologic symptom severity on any given day, we considered CFS and DLT separately, rather than in sum as the total PSH-AM. Additionally, given that DLT components include symptom persistence over 3 and 14 days, the exclusion of other etiologies, and the need for medications, confidence in diagnosis and stability of the DLT is likely to increase over the course of the initial two-week period. We therefore considered the DLT assessed on hospital day 14 as an outcome of interest.

### Group-Based Trajectory Modeling

The identification of distinct trajectories of physiologic data may illuminate underlying dynamic disease processes. While aggregated trajectories can obscure critical details, analyzing individual trajectories can become overly complex and muddled. GBTM advantageously balances detail with generalization[11]. GBTM is powered by statistical or machine learning methods to examine relationships among individual trajectories and classify them into subgroups with similar patterns. Consequently, trajectories within each subgroup exhibit more closely aligned trends than those in different subgroups. This data-driven approach is in contrast to that used in case-control or cohort studies, where patients are grouped according to some pre-determined classification. In order to illustrate this, we qualitatively compare CFS trend results using GBTM to those based on PSH case-control clinical diagnosis for patients included in this study. Trajectories based on clinical PSH diagnosis are illustrated in Supplementary Fig. 1.

We used unsupervised K-means clustering[18, 19] to conduct GBTM on a series of daily CFS scores in order to group patients with similar trajectories. Similarity was measured based on the distance metric[20, 21]. Dynamic Time Warping (DTW), a robust and popular shape-based similarity measure, was used as the distance metric. DTW identifies the best time series alignment and thus is a more intuitive distance measure compared to the conventional Euclidean distance metric, while considering the temporal pattern[22–24]. To determine the number of groups to describe our data best, we first used the elbow method and then evaluated the groupings for clinical relevance and information content. The elbow method heuristically determines the number of groups or clusters inherent in the dataset by plotting within-group error as a function of the number of groups and picking the “elbow” point of the curve, i.e., the point where adding an additional group starts to see diminishing returns of error reduction[25].

To infer clinical meaning from trajectory group membership, we explored the association between trajectory groups and outcomes. For the primary outcome, we focus on whether a clinical diagnosis of PSH was made (PSH case/control status). As a secondary more granular outcome of PSH diagnosis likelihood, we also evaluated relationships between trajectory group membership and DLT score. For other secondary outcome variables, we explored hospital discharge GCS scores (both total and component scores), ICU and hospital length of stay, mortality, and number of days on mechanical ventilation. Analysis of Variance (ANOVA) for continuous variables and the Chi-squared test for categorical variables were used to conduct these tests.

To explore the predictive utility of admission characteristics on trajectory group membership, we first performed univariate analyses, followed by multivariate multinomial logistic regression with forward stepwise selection for all the features with p-values less than 0.05 according to the univariate screening.

## Results

### Patient Cohort Characteristics

Table 1 summarizes descriptive statistics of the patient cohort, including demographic information, admission characteristics, injury details, radiographic features, and clinical outcomes.

### Trajectory Groups and Characteristics of Group Level Mean CFS Trends

Using the clustering method described above, we found diminishing returns of error reduction with more than five groupings. We then explored various models with respect to the number of groups (e.g., K = 3, 4, or 5), which were further evaluated for clinical relevance. We concluded that a four-group model provides the best tradeoff between granularity of information and depth of clinical insights. We report the four-group model results here.

**Figure 3:**
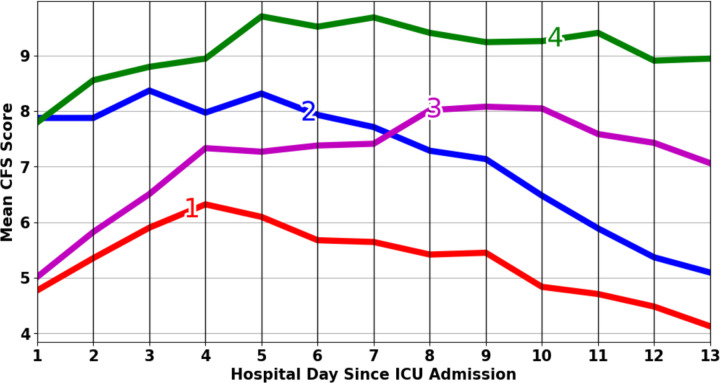
Distinct physiologic trajectories resulting from cluster analysis Mean daily CFS scores are displayed aggregated across patients belonging to each of four trajectory groups, represented by color. Each trajectory group demonstrates a distinct trend in CFS scores: Group 1 with persistently low scores (red, 14.03%, N=31), Group 2 with a decreasing score trend (blue, 33.03%, N=73), Group 3 with an increasing score trend (purple, 28.50 %, N=63), and Group 4 with persistently high scores (green, 24.43%, N=54). CFS: Clinical feature score of the paroxysmal sympathetic hyperactivity – assessment measure; ICU: intensive care unit.

Figure 3 summarizes the mean CFS trajectories aggregated from patients in each group. Group 1 is characterized by persistently low CFS scores. In contrast, patients in Group 3 start with similar scores to Group 1 but increase to moderate-high scores. Patients in Group 2 and Group 4 start with similarly high scores, but their trends diverge, with Group 4 demonstrating increasing and persistently high scores and Group 2 demonstrating scores that decline to lower levels.

### The Relationships Between Trajectory Group Membership and Outcomes

**Figure 4:**
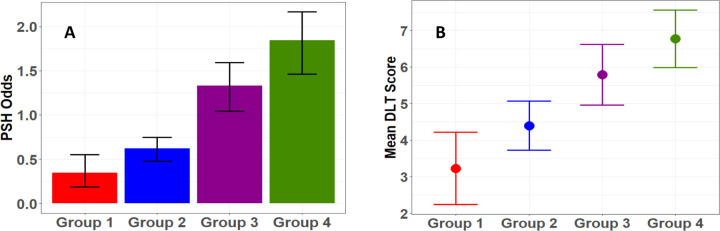
Relationships between physiologic trajectory group membership and PSH diagnosis Associations between trajectory group membership and PSH-related outcomes are displayed. Odds of PSH clinical diagnosis according to trajectory group membership are shown in subplot A, and the mean DLT score according to trajectory group is shown in subplot B, both with mean ±95% confidence interval. PSH: Paroxysmal Sympathetic Hyperactivity; DLT: Diagnosis Likelihood Tool.

We identified a significant association between CFS trajectory grouping and PSH diagnosis and DLT score (Table 2). Figure 4(a) summarizes the odds of PSH by group membership, while Fig. 4(b) displays mean DLT scores by group. As observed from these figures, patients in Groups 1 and 2 have significantly lower PSH odds (less than 1) and DLT scores compared to those in Groups 3 and 4. As far as the other outcome variables are concerned, we observe that group membership is significantly associated with ICU length of stay and number of days on mechanical ventilation (Table 2). Consistent with the association patterns noted with PSH diagnosis, patients in Groups 3 and 4 experienced significantly longer ICU stays and more days on mechanical ventilation compared to those in Groups 1 and 2. No significant association is found with hospital discharge GCS nor mortality.

### Admission Characteristics as Predictors for Trajectory Groups

Detailed results of univariate analyses of the relationships between admission characteristics and trajectory group membership are listed in Table 3. Age and body mass index (BMI) were shown to have a statistically significant association with trajectory group membership. Group 1 (persistently low CFS) demonstrated the oldest mean age of 52 years, while Group 4 (persistently high CFS) demonstrated the youngest mean age of 38 years. Group 4 also demonstrated the highest (but most variable) BMI of the four trajectory groups. A subset of the clinical features show significant relationships with groups, which includes initial total GCS, motor GCS (mGCS), and whether Intracranial pressure (ICP) monitoring is used. Specifically, Group 4, with a persistently high CFS trend and higher odds of PSH, had the lowest median GCS and mGCS scores and had the highest odds of receiving ICP monitors. Among the radiographic features, only intraventricular hemorrhage exhibited a significant association with trajectory group membership, with the highest rate occurring in Group 3.

The results of the final multinomial logistic regression model identifying admission characteristics that are independently predictive of group membership are presented in Table 4. Using stepwise regression with forward selection, statistical significance was observed for age and mGCS only.

## Discussion

Data-driven patient clustering represents a promising emerging approach to break down heterogeneity in TBI based on underlying mechanistic processes rather than relying on clinical constructs[26–28]. Using unsupervised K-means clustering and trajectory group analysis, we discovered four distinct CFS trend patterns during the first two weeks of acute TBI hospitalization. Beyond its relevance toward understanding the evolution of the specific clinical syndrome of PSH, this work represents a first step toward the identification of naturally-occuring autonomic endotypes in critically ill TBI patients. As such, the observed trajectory patterns demonstrate more nuance and divergence than those comparing clinically diagnosed PSH cases and controls (Fig. 3 vs Supplementary Material Fig. 1) and are worthy of further exploration.

Observed trajectory patterns differ in both initial CFS scores and evolution thereafter. We note that the trends following the initial days are more critical in predicting PSH diagnosis and DLT scores at day 14 than the initial CFS scores. Specifically, if the CFS trend does not decrease after 4 or 5 days, there is a higher likelihood of a positive PSH diagnosis. In contrast, a downward trend in CFS after the initial period correlates with a lower probability of PSH. The analysis suggests that CFS trends hold more promise as early predictors of PSH compared to initial CFS scores. Future work is warranted to identify the precise timing and trend features for the earliest reliable prediction of PSH. Correlation analysis between trajectory groups and outcome variables beyond PSH diagnosis demonstrated significant relationships with ICU LOS and number of days on mechanical ventilation. Therefore, early prediction and successful management of PSH could alter these outcomes, improving healthcare resource utilization and reducing clinical complications associated with longer ICU LOS and duration of mechanical ventilation. We did not observe significant relationships between trajectory group membership and other outcomes including mortality and hospital discharge GCS. We believe that this lack of observed effect may have been due to our relatively small, homogenous sample size of severe TBI patients who were hospitalized for at least 14 days, with selection bias leading to low mortality, and lack of long-term follow-up outcome data leading to fairly homogenous and crude outcomes. It remains unknown whether longer term functional outcomes differ according to sympathetic activation trajectory groups.

Exploring the relationships between admission characteristics and physiologic trajectory groups sheds light on potential predictors of PSH and naturally-occurring phenotypes of sympathetic nervous system activation following acute TBI. Univariate screening identified a subset of baseline and early admission variables significantly associated with trajectory group membership, including age, BMI, tGCS, mGCS, and use of invasive ICP monitoring. In the subsequent fitting of the multivariate multinomial logistic regression model, only two variables were found to be relevant: age and mGCS. Specifically, the odds of belonging to high CFS groups (3 & 4) are decreased by 3% and 4% for each increase of one year in age after controlling for mGCS score. This observation aligns with previous literature suggesting that younger age is associated with a higher risk of PSH in adults[17, 29, 30]. Interestingly, the opposite effect has been observed in pediatric populations, where older age is associated with greater risk for PSH[31]. Rather than a simple ordinal risk factor, age may therefore be an important grouping factor for physiologic trajectories across the entire span of neurodevelopment and natural aging. We also found that for each unit decrease in mGCS, the odds of belonging to group 4 (persistently high CFS and increased risk for PSH) increased by 9%. This is consistent with published literature suggesting that lower initial GCS increases the risk for PSH[32].

Given that the CFS score is composed of clinical and physiologic markers of sympathetic nervous system activation, CFS trajectory groups may provide insight beyond prediction of the syndrome of PSH, extending into the drivers and evolution of post-TBI dysautonomia, more generally. Following TBI, sympathetic activation has been associated with higher injury severity, increased mortality, and mechanisms of secondary injury including inflammation, coagulopathy, endothelial dysfunction, and glymphatic system dysfunction[33–36]. Further, there is evidence to suggest that interventions that reduce sympathetic activation and its end-organ consequences block these effects[36–38]. However, many features related to sympathetic activation including its timing, magnitude, persistence and pharmacologic modulation, may contribute to its effects on secondary injury mechanisms and outcomes. As a persistent but paroxysmal phenomenon, it is unclear whether PSH specifically contributes to these sympathetically mediated effects, or whether alternative autonomic phenotypes should be considered as targets for interventions to improve outcomes. Nonetheless, the PSH-AM score provides a framework for quantifying sympathetic hyperactivity via the CFS score and may serve as a starting point for phenotyping post-TBI dysautonomia in a standard way.

This study represents preliminary work with a number of limitations beyond those already discussed. First, CFS trends were derived from retrospectively allocated CFS scores based on EHR review of vital signs and clinical documentation. Prospective documentation of PSH episodes and analysis using raw vital signs may provide more accurate and nuanced physiologic information. We used a convenience sample of critically ill TBI patients who were scored according to the PSH-AM as part of prior work[7, 39, 40]; as a whole our included cohort was younger with more severe injuries compared to the held out eligible patients (Supplementary Material Table 1) and therefore may have been at higher risk for post-TBI dysautonomia.

Larger, multi-center prospective studies are needed to confirm and expand upon these findings. Larger datasets will generate more power to identify baseline, clinical, and outcome variables associated with each trajectory group. For example, we demonstrated trend-level associations between initial CT radiographic features and trajectory group membership, which could become significant with more statistical power. Stronger phenotypic descriptions of the trajectory groups may further suggest heterogeneous mechanistic targets for autonomic nervous system targeted therapies in traumatic brain injury.

## Conclusion

This study explored the heterogeneous and dynamic trends of PSH-AM CFS scores among critically ill adult TBI patients, which adds to the sparse literature characterizing temporal trends of sympathetic nervous system activation following TBI. Our work provides potentially useful insights for clinicians managing dysautonomia, generally, and PSH, more specifically, during the acute phase following TBI. We demonstrated significant associations between various baseline and early admission features, trajectory group membership, eventual PSH diagnosis, and duration of ICU stay and mechanical ventilation. Early recognition of a patient’s physiological trajectory may lead to earlier diagnosis and management of PSH and other phenotypes of dysautonomia following TBI. This may open avenues toward tailored interventions for limiting secondary injury and improving TBI recovery.

## Figures and Tables

**Table 1:** Included patient characteristics

Demographics	
Age, years	44 (19)
BMI	27 (11)
Sex	Male: 175 (79)
Race	White: 115 (52); Black: 80 (36); Others: 26 (12)
Ethnicity	Not Hispanic/Latino: 194 (88)
**Injury Characteristics**	
Non-penetrating injury	202 (91)
Injury Severity Score	26 (19, 34)
Head AIS	4 (3, 5)
Trauma injury severity score	0.75 (0.48, 0.90)
**Admission Clinical Characteristics**	
GCS score on ICU admission	
- Eye	1 (1, 3)
- Motor	4 (2, 5)
- Verbal	1 (1, 1)
- Total	7 (4, 9)
Following commands on ICU admission	49 (22)
Craniectomy/Craniotomy	68 (31)
Intracranial Pressure Monitor Placed	120 (54)
**Initial CT Radiographic Characteristics**	
Marshall Score	2 (2, 4)
Total Rotterdam CT score	3 (3, 4)
- Compression of basal cisterns	Partial: 52 (24); Complete: 22 (10)
- Midline shift of > or = 5mm	59 (27)
- Absence of epidural hemorrhage	198 (90)
- Presence of Intraventricular or subarachnoid hemorrhage	163 (74)
Contusion	105 (48)
Intraventricular hemorrhage	43 (19)
Hydrocephalus	21 (10)
Extra-axial hemorrhage	142 (64)
Diffuse axonal injury	36 (16)
**Hospital Discharge Outcome**	
Mortality, Survived, n (%)	207 (94)
GCS at hospital discharge	
- Eye	4 (4, 4)
- Motor	6 (5, 6)
- Verbal	3 (1, 5)
- Total	13 (10, 15)
Following commands at hospital discharge	160 (72)
Hospital LOS, days	29 (14)
ICU LOS, days	20 (10)
Days on Ventilation	14 (9)
DLT	4 (2, 8)

Continuous variables are expressed as mean (SD). Ordinal or non-normally distributed continuous variables are expressed as median (25th, 75th percentile). Categorical variables are expressed as count (percentage). AIS: Abbreviated Injury Score; BMI: Body Mass Index; DLT: Diagnostic Likelihood Tool score; GCS: Glasgow Coma Scale; ICU: Intensive Care Unit; LOS: Length of Stay.

**Table 2:** Relationships between physiologic trajectory group membership and in-hospital outcomes

Variable	Group 1	Group 2	Group 3	Group 4	P-value
*N*	*31*	*73*	*63*	*54*	
PSH Case/Control, n (%)					**< 0.001**
PSH(+)	8 (25.81%)	28 (38.36%)	36 (57.14%)	35 (64.81%)	
PSH(−)	23 (74.19%)	45 (61.64%)	27 (42.86%)	19 (35.19%)	
DLT	2 [1, 4]	4 [2, 7]	7 [2, 9]	7.5 [4, 9]	**< 0.001**
Mortality, Survived	28 (90.32%)	71 (97.26%)	56 (88.89%)	52 (96.29%)	0.16
GCS at Hospital Discharge					
- Eye	4 [4, 4]	4 [4, 4]	4 [4, 4]	4 [4, 4]	0.36
- Motor	6 [5, 6]	6 [5, 6]	6 [5, 6]	6 [5.25, 6]	0.95
- Verbal	2 [1, 4]	4 [1, 5]	1 [1, 4.5]	3 [1, 4]	0.23
- Total	12 [10.5, 14]	14 [11, 15]	11 [10, 14.5]	12.5 [11, 14]	0.45
Following commands at Hospital Discharge, n (%)	22 (73.33%)	56 (78.87%)	42 (72.41%)	40 (76.92%)	0.83
Hospital LOS, days	27.58 (11.13)	26.98 (11.52)	29.73 (16.45)	31.3 (15.53)	0.092
ICU LOS, days	17.78 (10.47)	17.54 (8.90)	21.11 (11.08)	21.5 (11.05)	**0.019**
Days on Ventilation	12.65 (9.91)	11.29 (8.00)	15.02 (9.88)	15.28 (9.42)	**0.024**

ANOVA test results on the association between trajectory groups and outcome variables (p-value < 0.05 are bold faced). Continuous variables are expressed as median [25th, 75th percentile], or as mean (SD); Categorical variables are expressed as count (percentage). DLT: Diagnostic Likelihood Tool score; GCS: Glasgow Coma Score; ICU: Intensive Care Unit; LOS: Length of Stay.

**Table 3:** Relationships between baseline and early admission features with physiologic trajectory group membership

Variable	Group 1	Group 2		Group 3	Group 4	P value
Sex, n (%)						0.30
Male	24 (77.42%)	56 (76.71%)		55 (87.30%)	40 (74.07%)	
Female	7 (22.58%)	17 (23.29%)		8 (12.69%)	14 (25.93%)	
Race, n (%)						0.42
White	17 (54.84%)	42 (57.53%)		30 (47.62%)	26 (48.15%)	
Black	10 (32.26%)	24 (32.88%)		24 (38.09%)	22 (40.74%)	
Other	4 (12.90%)	7 (9.59%)		9 (14.29%)	6 (11.11%)	
Age, years	51.71 (19.33)	48.25 (19.18)		40.24 (18.45)	37.87 (14.94)	**< 0.001**
BMI	25.14 (7.28)	25.52 (6.82)		26.23 (4.84)	30.73 (18.99)	**0.010**
Injury Type, n (%)						0.84
Blunt	28 (90.32%)	66 (90.41%)		57 (90.48%)	51 (94.44%)	
Penetrating	3 (9.68%)	7 (9.58%)		6 (9.52%)	3 (5.56%)	
Injury Severity Score	26 [18, 30]	26 [17, 30]		26 [21, 33.5]	26 [22, 34]	0.55
Head AIS	4 [3, 5]	4 [3, 5]		4 [3, 5]	4 [3, 5]	0.68
Following Commands at ICU admission, n(%)	5 (16.13%)	20 (27.39%)		11 (17.46%)	13 (24.07%)	0.43
Had Brain Surgery Performed, n (%)	10 (32.26%)	29 (39.73%)		16 (25.39%)	13 (24.07%)	0.19
Intracranial Pressure Monitor Placed, n (%)	16 (51.61%)	29 (39.73%)		38 (60.32%)	37 (68.52%)	**0.009**
Marshall score	2 [2,4.5]	2 [2, 5]		2 [2, 3]	2 [2, 3]	0.11
Total Rotterdam CT score	3 [3, 4]	3 [3, 4]		3 [3, 4]	3 [3, 3]	0.16
- Compression of Basal Cisterns, n (%)						0.26
Partial	7 (22.6%)	23 (31.5%)	14 (22.2%)		8 (14.8%)	
Complete	4 (12.9%)	7 (9.6%)	8 (12.7%)		3 (5.7%)	
- Midline Shift of > or = 5mm, n (%)						
Yes	9 (29.03%)	26 (35.62%)	12 (19.05%)		12 (22.22%)	0.14
- Epidural Hemorrhage, n (%)						
Absent	29 (93.55%)	65 (89.04%)	66 (88.89%)		49 (89.79%)	0.71
- Intraventricular or Subarachnoid Hemorrhage, n (%)						
Present	24 (77.42%)	49 (67.12%)	48 (76.19%)		42 (77.78%)	0.47
Contusion, n (%)	17 (54.84%)	35 (47.95%)	33 (52.38%)		20 (37.04%)	0.30
Intraventricular Hemorrhage, n (%)	7 (22.58%)	7 (9.59%)	18 (8.57%)		11 (20.37%)	**0.044**
Hydrocephalus, n (%)	4 (12.90%)	6 (8.22%)	9 (14.29%)		2 (3.70%)	0.23
Extra-Axial Hemorrhage, n (%)	24 (77.42%)	51 (69.86%)	34 (53.97%)		33 (61.11%)	0.09
Diffuse Axonal Injury, n (%)	2 (6.45%)	9 (12.33%)	15 (23.81%)		10 (18.52%)	0.12
GCS at Trauma Admission						
- Total	7 [3.5,13]	7 [3, 13]	6 [3, 8]		4 [3,7.75]	**0.002**
- Motor	4 [1.5,6]	5 [1, 6]	3 [1, 5]		2 [1, 4]	**< 0.001**
Trauma Injury Severity Score	0.77 [0.50,0.88]	0.78 [0.51,0.93]	0.7 [0.46,0.87]		0.75 [0.49,0.90]	0.64
GCS at ICU Admission						
- Eye	1 [1, 2]	1 [1, 3]	1 [1, 2]		1 [1, 2]	0.74
- Verbal	1 [1, 1]	1 [1, 2]	1 [1, 1]		1 [1, 1]	0.52
- Motor	4 [1, 5]	5 [4, 6]	4 [1, 5]		4 [3, 5]	0.31
-Total	7 [3,8.5]	7 [6, 11]	6 [3, 7]		6.5 [5,8.75]	0.73

Univariate screening for significant association between trajectory groups and demographics, admission & clinical characteristics (p-value < 0.05 are bold faced). AIS: Abbreviated Injury Score; BMI: Body Mass Index; GCS: Glasgow Coma Score; ICU: Intensive Care Unit. Continuous variables are expressed as median (25th, 75th percentile), or as mean (SD); categorical variables are expressed as count (percentage).

**Table 4:** Independent predictors of physiologic trajectory group membership

Predictor	Odds Ratio (OR)	95% CI		P value
		Lower	Upper	
*age*				
Group 2	0.99	0.97	1.01	0.37
**Group 3**	**0.97**	**0.95**	**0.99**	**0.014**
**Group 4**	**0.96**	**0.94**	**0.99**	**0.004**
*mGCS at trauma admission*				
Group 2	1.04	0.83	1.29	0.74
Group 3	0.87	0.69	1.09	0.21
**Group 4**	**0.81**	**0.64**	**1.00**	**0.009**

Multivariate multinomial logistic regression results in association between trajectory groups and admission characteristics; odds ratio are expressed in comparison to Group 1 as the baseline group.
